# A multi-kernel and multi-scale learning based deep ensemble model for predicting recurrence of non-small cell lung cancer

**DOI:** 10.7717/peerj-cs.1311

**Published:** 2023-05-02

**Authors:** Gihyeon Kim, Young Mi Park, Hyun Jung Yoon, Jang-Hwan Choi

**Affiliations:** 1 Department of Computational Medicine, Graduate Program in System Health Science and Engineering, Ewha Womans University, Seoul, South Korea; 2 Department of Molecular Medicine, College of Medicine, Ewha Womans University, Seoul, South Korea; 3 Department of Radiology, Veterans Health Service Medical Center, Seoul, South Korea; 4 Division of Mechanical and Biomedical Engineering, Graduate Program in System Health Science and Engineering, Ewha Womans University, Seoul, South Korea; 5 Department of Artificial Intelligence, Ewha Womans University, Seoul, South Korea

**Keywords:** Multi-scale network, Multi-kernel network, Ensemble model, Non-small cell lung cancer, Lung cancer recurrence, Artificial neural network, Deep learning

## Abstract

Predicting recurrence in patients with non-small cell lung cancer (NSCLC) before treatment is vital for guiding personalized medicine. Deep learning techniques have revolutionized the application of cancer informatics, including lung cancer time-to-event prediction. Most existing convolutional neural network (CNN) models are based on a single two-dimensional (2D) computational tomography (CT) image or three-dimensional (3D) CT volume. However, studies have shown that using multi-scale input and fusing multiple networks provide promising performance. This study proposes a deep learning-based ensemble network for recurrence prediction using a dataset of 530 patients with NSCLC. This network assembles 2D CNN models of various input slices, scales, and convolutional kernels, using a deep learning-based feature fusion model as an ensemble strategy. The proposed framework is uniquely designed to benefit from (i) multiple 2D in-plane slices to provide more information than a single central slice, (ii) multi-scale networks and multi-kernel networks to capture the local and peritumoral features, (iii) ensemble design to integrate features from various inputs and model architectures for final prediction. The ensemble of five 2D-CNN models, three slices, and two multi-kernel networks, using 5 × 5 and 6 × 6 convolutional kernels, achieved the best performance with an accuracy of 69.62%, area under the curve (AUC) of 72.5%, F1 score of 70.12%, and recall of 70.81%. Furthermore, the proposed method achieved competitive results compared with the 2D and 3D-CNN models for cancer outcome prediction in the benchmark studies. Our model is also a potential adjuvant treatment tool for identifying NSCLC patients with a high risk of recurrence.

## Introduction

Lung cancer remains the leading cause of cancer-related mortality despite advancements in cancer research, and it is the second most common cancer apart from prostate and breast cancer incidence (Wankhede, 2021). Non-small cell lung cancer (NSCLC) is the most common subtype, accounting for 85% of lung cancer diagnoses globally (Sève, Reiman & Dumontet, 2010). Surgery is the standard treatment for patients with NSCLC. However, high recurrent rates after resection have been the most difficult challenge for completely removing and disseminating cancer cells during surgery (Hashimoto et al., 2014; Hayashi et al., 1999; Uramoto & Tanaka, 2014). Therefore, NSCLC is associated with poor survival rates even after diagnosis in the early stages.

The tumor, node, and metastasis classification (Mountain, 1997) is a staging system and a powerful prognostic tool for predicting the survival rates in patients with NSCLC (Brundage, Davies & Mackillop, 2002). This classification also plays a vital role in determining treatment strategies, such as adjuvant chemotherapy and radiotherapy (Katsumata et al., 2019; Postmus et al., 2017). Therefore, it is vital to classify patients into the correct clinical stage. However, several issues still exist regarding this strategy, and a wide variation still exists in the recurrence incidence after curative resection in patients at the same clinical stage (Uramoto & Tanaka, 2014). Moreover, the current nodal classification has several problems because of the prognostic heterogeneity of patients in the nodal stage and the necessity of rearrangement of the nodal staging system (Caldarella et al., 2006; Katsumata et al., 2019; Osaki et al., 2004; Yano et al., 1994).

Recent studies have used deep-learning models to predict the prognosis of various cancers (Afshar et al., 2020b; Hosny et al., 2018; Huang, Xue & Wu, 2019; Li et al., 2017; Xiao et al., 2018). Typically, the convolutional neural network (CNN) is the most representative method used for cancer outcome prediction based on medical images. Li et al. (2017) used deep learning-based radiomics to predict noninvasive IDH1 for low-grade glioma. Moreover, CNN was used to segment tumors in magnetic resonance images, and image features were obtained by normalizing the information of the last convolutional layers. Their study found that deep learning-based radiomics, based on multi-modality magnetic resonance images, improved the area under the curve (AUC) to 95% and outperformed the traditional radiomics method. Hosny et al. (2018) predicted the two-year overall survival rate of lung cancer in surgery patients, using three-dimensional (3D) CNN. The prognostic signatures were identified using 3D CNN for patients treated with radiotherapy, and transfer learning was employed to achieve the same for surgery patients. Although these models were successful in predictions of cancer outcomes, the predictions were limited to features extracted on a specific scale.

Recent studies have demonstrated the effectiveness of the ensemble method in overcoming these shortcomings. Huang, Xue & Wu (2019) proposed a new amalgamated-CNN framework to predict pulmonary nodules based on the ensemble learning of the 3D CNNs. The networks used three input sizes: 32 × 32 × 32, 64 × 64 × 64, and 96 × 96 × 96, and the results of these three CNNs were fused using an adaptive boosting classifier for better prediction. Xiao et al. (2018) applied a multi-model ensemble method, using deep learning to incorporate the outputs of five machine learning models. Afshar et al. (2020b) developed a multi-scale framework for survival prediction in pulmonary malignancies, using positron emission tomography (PET)/computed tomography (CT) images and other clinical tools. The framework consists of two parallel CNNs, trained on three slices of various scales of CT and PET images. The outputs of the two CNNs and other clinical factors were processed using a random survival forest for the final prediction. This study revealed that multi-scale networks outperform their single-scale counterparts in overall survival prediction.

Most studies on ensemble models for cancer prediction have focused on the features extracted using multiple 3D CNN models (Afshar et al., 2020b; Huang, Xue & Wu, 2019). Despite the abundant information extracted from the multi-scale 3D models, these models have failed to employ features extracted from diverse model architectures, using the same image input.

This study proposes a multi-model ensemble-based prediction for two-year recurrence after surgical resection in patients with NSCLC. The proposed model is an ensemble architecture using a combination of results from various 2D CNN models, which vary in input images and model architectures. Multiple inputs, including five slices spaced five mm apart and two multi-scale inputs, were used to extract features from various 2D slices. Furthermore, models of different convolutional kernel sizes were used on the same input to extract features from various model architectures. The integration of prediction values was processed through a set of a fully connected network to ensemble multiple 2D CNN models. Several combinations of models were evaluated to determine the best ensemble of multi-scale and multi-kernel networks. We benchmarked the model performance against models built on single-scale and 3D inputs. We compared the ensemble approach with other methods, such as machine learning and majority voting. The final results indicate that the ensemble of multi-scale inputs and multi-kernel networks allows feature extraction from the tumor region and surrounding tissues. Moreover, the deep learning-based ensemble approach leads to a more accurate prediction than machine learning or majority voting algorithm.

The novel contributions of this work are fourfold: First, we developed a deep ensemble model based on pretreatment CT images to predict early recurrence in patients with NSCLC. This deep learning-based ensemble approach improved the overall model performance by successfully synthesizing the predictions of several models. Second, multiple 2D slices were used instead of single-slice or 3D inputs to capture information from the major CT slices of each patient. Third, multi-scale and multi-kernel networks assist our framework to capture features from the tumor region and surrounding tissues. Finally, we compared multiple ensemble models to determine the best combination of 2D CNN models with varied inputs and model architectures.

## Materials and Methods

The dataset was a collection of 530 patients with NSCLC. The average age of the patients was 72.22 (range: 43–93), and these patients were diagnosed with lung adenocarcinoma or lung squamous cell carcinoma and had undergone CT before surgical resection. Table 1 provides the demographic and clinical characteristics of patients with NSCLC. The data were gathered from the VHS Medical Center and The Cancer Imaging Archive (Clark et al., 2013) databases, in which the data comprised clinical data and CT images. Any patients with a history of multiple surgeries were excluded, and images acquired using the GE or SIEMENS CT scanner were used. The tumors in the CT images were manually annotated by two radiologists, with more than 10 years of experience in lung diagnosis. The VHS Medical Center Institutional Review Board (IRB) approved the study protocol (IRB No. BOHUN-2018-09-015). The Cancer Imaging Archive database is publicly available, and IRB approval is not required.

**Table 1:** Demographic and clinical characteristics of patients with NSCLC from the VHS Medical Center and The Cancer Imaging Archive.

	Number of Patients (%)	
Institution		
VHS Medical Center	406	(76.6%)
TCIA (R01)	124	(23.4%)
Age		
Age <70	174	(32.8%)
Age ≥ 70	356	(67.2%)
Histology		
Adenocarcinoma	291	(54.9%)
Squamous cell carcinoma	239	(45.1%)
Recurrence	267	(50.4%)
Early recurrence (<2 years)	208	(77.9%)
Late recurrence (≥2 years)	59	(22.1%)
No recurrence	253	(49.6%)

### Preprocessing

Multiple studies have demonstrated that “CT scanner variability” adversely influences extracting features (Chang et al., 2013; Mackin et al., 2015). Only the CT scans acquired using GE and Siemens CT scanners were used to reduce the influence of inter-scanner variability in the classification task.

**Figure 1:**
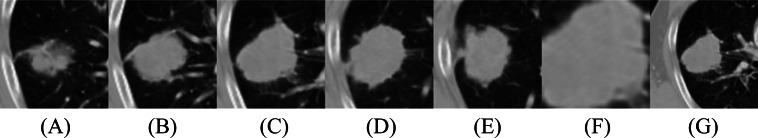
Illustration of CT images for model input: (A–E) Five slices of 100 × 100 and (F, G) two multi-scale images, 50 × 50 and 150 × 150. (F) and (G) were taken from the same 2D slice as (C), the central tumor slice.

The proposed deep learning model cropped CT images into 2D patches of various slices or scales, as depicted in Fig. 1. For each patient, pixel values were converted into Hounsfield units and resampled into isotropic voxels to remove variance in slice thickness and in-plane resolution, where one voxel corresponded to 1 × 1 × 1 mm (Xu et al., 2019). Afterward, 3D isotropic patches were normalized into a 0–255 range, using lower and upper Hounsfield unit bounds of −1,000 and 400 to fit the digital grayscale image format. Finally, the images were resized into 512 × 512 to match the size of the original image (Li et al., 2021). Each 2D input was extracted from five slices and three scales. The performance is improved by increasing the number of scales, and this allows the model to capture more detailed inter- and intra-tumoral heterogeneity. However, using three scale patches is the standard approach to reduce memory usage and computing time (Afshar et al., 2020a; Afshar et al., 2020b; Tafti et al., 2018). Smaller patches provide better consistency but fail to retain the similarity with the whole object. Moreover, large scales cannot capture object details (Hao et al., 2012).

In this study, we used multi-scale inputs of 50 × 50, 100 × 100, and 150 × 150, representing information from fine, medium, and coarse nodule boundaries, respectively (Afshar et al., 2020b; Chaddad et al., 2018). The second scale (100 × 100) fits the majority part of the tumor boundary. Five patches were cropped from the central tumor slice, two slices before the central tumor slice, and two slices after the central tumor slice. The slices had a 5-mm interval between axial slices because 5-mm was the maximum slice thickness of the CT images, without considering outlier values, deviating more than two standard deviations to either side of the mean. The outliers include slices with substantial slice thickness but are small in number. The central tumor slice is the slice with the maximum tumor area within a patient’s 3D CT volume. The 100 × 100 2D patches were extracted around each center of mass, capturing 90.6% of the tumor-bounding box dimensions in the dataset. These five patches are called the *second before slice*, *first before slice*, *medium slice*, *first after slice*, and *second after slice*. The third scale was extracted by allowing a margin of 25 pixels on each side for 150 × 150 and was down-sampled to 100 × 100, using bi-cubic interpolation. The first scale was extracted with a size of 50 × 50 and was up-sampled to 100 × 100, using bi-cubic interpolation. For data augmentation, random horizontal and vertical flipping was performed in real-time during training to enhance the diversity of the training samples.

### Proposed ensemble prediction model

#### 2D-convolutional neural network models

**Figure 2:**
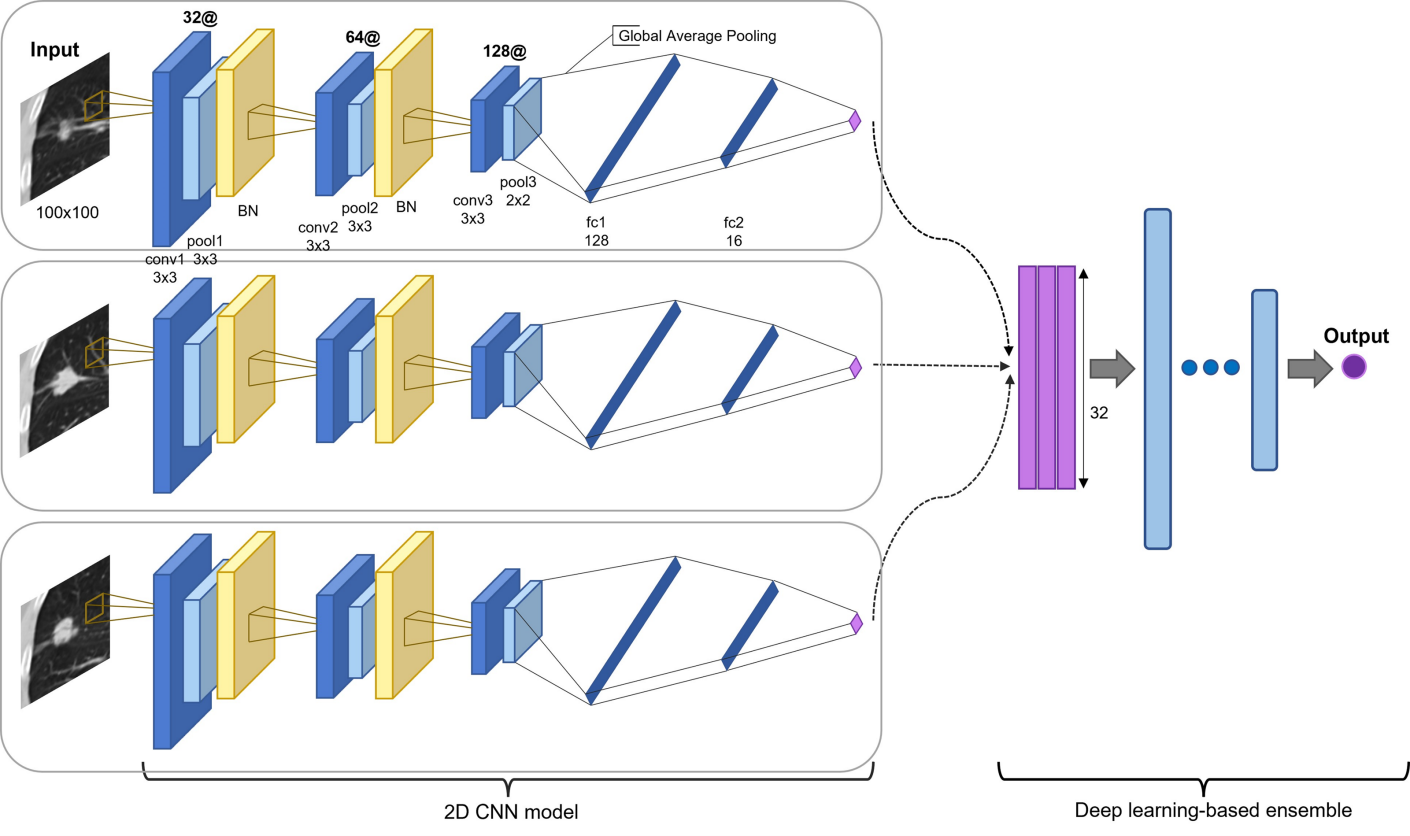
Proposed model architecture. Two-dimensional CNN models are based on different input slices, multi-scale inputs, and multi-kernel networks. The output vectors are concatenated and processed through a set of fully connected layers for the final prediction (recurred or not recurred). The example presents the assembly of 2D CNN models based on three slices: the first before slice, the medium slice, and the first after slice.

The proposed model is an ensemble model of multiple 2D-CNNs, broadly divided into two model architectures: multi-scale and multi-kernel networks. Figure 2 presents the overall algorithm. All images are cropped or resized into 100 × 100 for the input layer, followed by three consecutive convolutional blocks. The convolutional block consists of a convolutional layer, a max-pooling layer, and a parametrized rectified linear unit (PReLU) (He et al., 2015), stacked one after another. The first and second max-pooling layers used 3 × 3 filters, and the last pooling layer used a 2 × 2 filter. The last pooling layer applied a smaller filter than the previous convolutional blocks to leave more useful features after global average pooling for the final prediction and to use the same filter for small-scale input. A batch normalization layer (Ioffe & Szegedy, 2015) was inserted after the first and second convolutional blocks.

The model was applied for the five slices, using a 3 × 3 convolutional kernel. Multi-scale inputs of 50 × 50 and 150 × 150 were resized to 100 × 100 and processed through a multi-scale network of the same architecture. Multi-kernel networks were further applied for the medium slice. *Multi-kernel networks* are models with different convolutional kernel sizes of 2 × 2, 4 × 4, 5 × 5, and 6 × 6 that are used to extract various features from the same input image. Following the convolutional blocks, the global average pooling operation calculates a global average value of all elements in the corresponding feature map. This layer can reduce training parameters to avoid over-fitting, and this is more robust to spatial translations of the input (Lin, Chen & Yan, 2013). Finally, the global average pooling layer is followed by two consecutive fully connected layers and followed by a final PReLu. The outcome was computed using sigmoid activation in determining the probability of the patient exhibiting an early recurrence of NSCLC. Most recurrences occur within the first two years. Thus, we used two years as the cutoff value (Higgins et al., 2012; Kiankhooy et al., 2014; Varlotto et al., 2013). The batch normalization layer plays a significant role in stabilizing the optimization problem and in reducing the internal covariate shift problem (Ioffe & Szegedy, 2015; Santurkar et al., 2018). Thus, we employed five-fold cross-validation to reduce the generalization error and prevent over-fitting (Anzai, 2012; Xiao et al., 2018).

#### Multi-model ensemble based on deep learning

**Figure 3:**
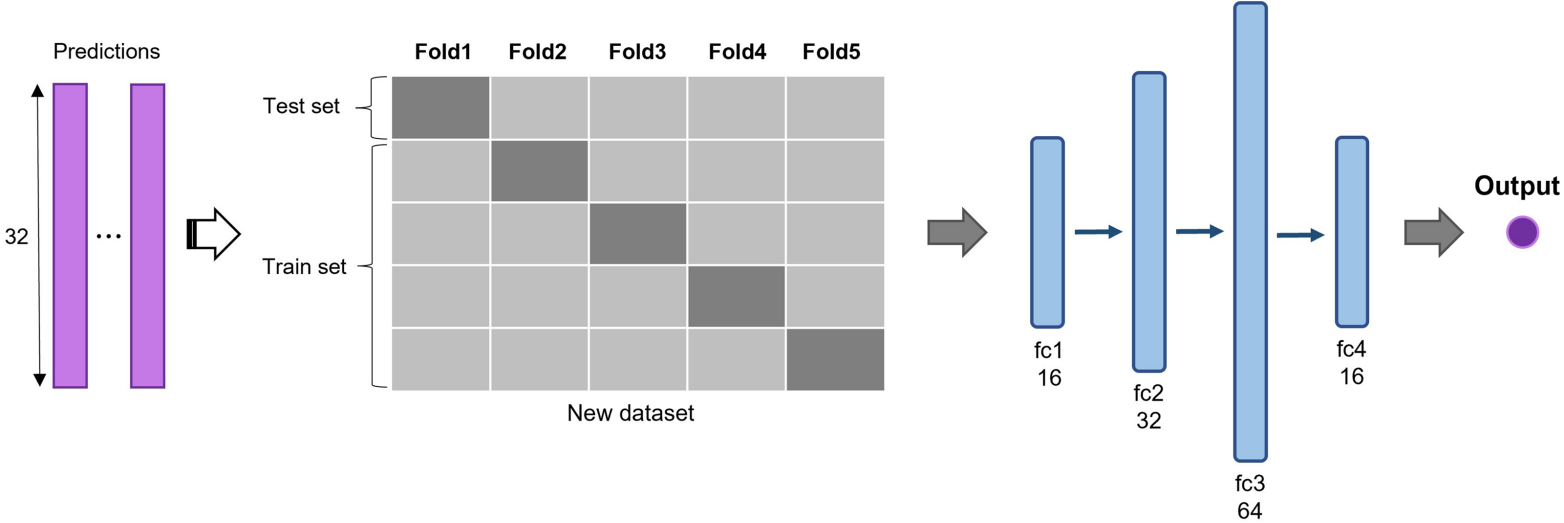
Deep learning-based ensemble method. Outputs of multiple CNNs are concatenated to form a new dataset and input into a set of fully connected layers for final prediction.

The predictions of 2D CNN models are assembled for the input layer of the multi-model ensemble approach. A total of three, five, and six models were ensembled to determine the best combination. The predictions of individual models are concatenated to form a new dataset and divided into five subsets (Fig. 3). The same five-fold cross-validation setting, used for the 2D CNN models, was employed to verify the robustness of the ensemble algorithm. We used a five-layer neural network to classify recurrent and non-recurrent tumor samples. Following the input layer, 16, 32, 64, and 16 neurons were applied to each layer. For nonlinear operation, PReLu was used after all four consecutive fully connected layers, and a final sigmoid function was used. The output class has one dimension with a value of 0 or 1, indicating a non-recurrent or recurrent tumor, respectively. The deep learning-based ensemble strategy can automatically learn complex relationships between multiple classifiers. Unlike the general ensemble strategy, such as majority voting algorithms and machine learning methods, this strategy considers non-linear relationships of various models.

### Implementation details

The network structure was implemented in Python, using the PyTorch software package (Paszke et al., 2019). The mini-batch of size 32 was used for all models to prevent over-fitting the training set. The number of epochs was set to 500 for each CNN model and 300 for the final ensemble model. The multi-model ensemble network was trained to minimize the binary cross-entropy loss. Stochastic gradient descent was employed as an optimization method, in which the learning rate started from 5 ×10^−3^ with a decay factor of 0.7 on every 50 epochs, and the momentum was 0.5.

### Performance metrics

We evaluated the proposed framework using the six most-used metrics to compare the prediction performance: accuracy, AUC, F1 score, precision, recall, and Mathew’s correlation coefficient (MCC) (Bukhari, Webber & Mehbodniya, 2022). Accuracy, denoted as the overall correctness, determines how many test samples are correctly classified. We used a balanced dataset, and 2D-CNN models with the best accuracy were selected. The AUC is a widely used metric for binary classification, reflecting the probability that a classifier ranks a randomly chosen positive example higher than a randomly chosen negative example. Furthermore, the F1 score is a harmonic mean of precision and recall, which refers to the precision and robustness of a classifier. Precision is defined as the fraction of correctly identified patients with NSCLC recurrence, and recall is measured as the proportion of predicted recurred patients to all relevant samples. The MCC is a performance metric that outputs a value between −1 and +1, where −1 represents total discordance; 0 represents no better than random predictions, and +1 represents perfect agreement between the actual observations and predictions. Through cross-validation, the mean values and standard deviation were calculated for the five test sets. These evaluation metrics were used to assess the performance of each 2D-CNN model and the multi-model ensemble networks.

## Results

### Performance of the 2D-CNN model for NSCLC recurrence prediction

**Figure 4:**
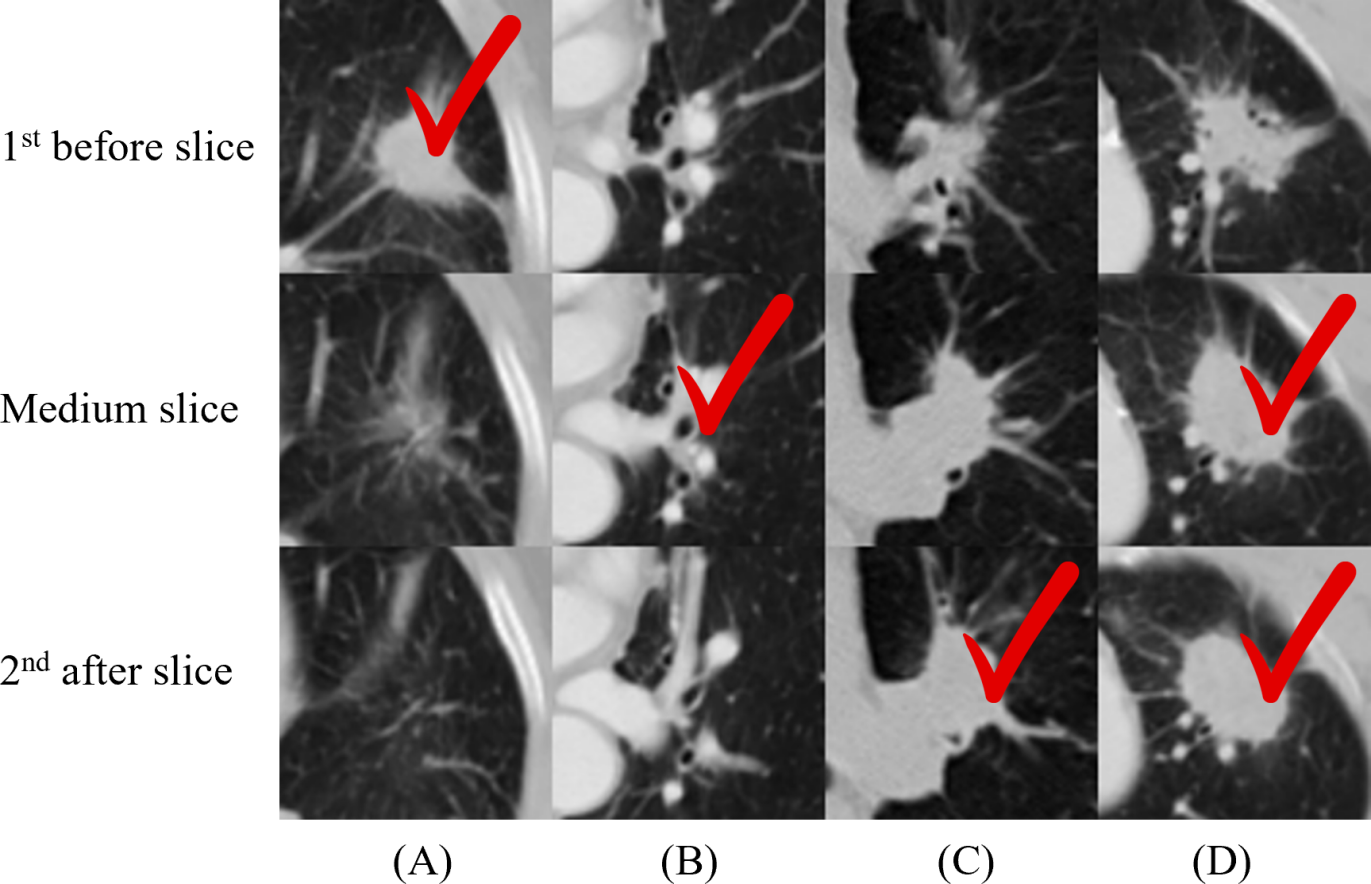
(A–D) Cases where not all the single-scale models provided correct predictions. One column indicates three slices from one nodule, and the check sign indicates the successful slices. For (A), only the first before slice made a correct prediction. This figure illustrates the necessity of including all three slices in the final model.

We applied 2D-CNN models on several inputs: five slices and two multi-scale inputs. A convolutional kernel size of 3 × 3 was applied for the model on all input types, and the sizes of 2 × 2, 4 × 4, 5 × 5, and 6 × 6 were applied for the medium slice to determine the influence of the kernel size on the final prediction. Table 2 summarizes the prediction results. The model with the 3 × 3 convolutional kernel on the medium slice was used as a baseline model. The results revealed that the multi-scale network applied on 150 × 150 input achieved the lowest accuracy of 66.04%. Other models demonstrated similar performance with the best accuracy of 68.87%, despite having different inputs and model architectures. Figure 4 illustrates the four nodules from three slices, which are the first before slice, the medium slice, and the first after slice. Figure 4 indicates the slice that successfully classifies tumor recurrence. This outcome reveals that all three slices are vital for accurate prediction, and one slice is challenging for representing information of on the 3D tumor volume.

**Table 2:** Overall performance comparison of 2D-CNN models. Models using five slices and multi-scale inputs were applied with 3 × 3 convolutional kernels, and multi-kernel networks were applied with varying convolutional kernel sizes of 2 to 6.

		Accuracy	AUC	F1 score	Precision	Recall	MCC
Five slices	2nd before	68.11	70.34	67.76	69.87	67.06	0.3705
	1st before	67.93	71.3	68.13	68.67	68.56	0.3643
	medium	67.93	71.6	68.13	68.97	68.95	0.3676
	1st after	**68.87**	71.5	**68.65**	70.3	67.82	0.3822
	2nd after	68.11	70.43	67.9	69.17	67.83	0.3696
Multi-kernel NW	2 × 2	68.11	71.19	68.19	69.67	68.18	0.3715
	4 × 4	68.68	**72.77**	68.52	**70.44**	68.56	**0.3839**
	5 × 5	67.74	72.54	67.81	68.56	67.81	0.3589
	6 × 6	67.93	72.19	68.38	68.55	**69.3**	0.3643
Multi-scale NW	50 × 50	67.55	71.22	67.11	68.46	66.32	0.3539
	150 × 150	66.04	68.99	66.97	66.42	68.15	0.3238

**Notes.**

Values in bold indicate the best-performing model for each evaluation metric.

### Performance of the proposed multi-model ensemble network

The proposed multi-model ensemble framework concatenated the predictions of three, five, and six models, as listed in Table 3. Several groups were evaluated to determine the best combination of multi-scale and multi-kernel networks with various input slices. As inferred from this Table 3, the integration strategy of multiple models significantly outperformed individual prediction models based on accuracy, F1 score, and precision.

**Table 3:** Overall performance comparison of ensemble models with various combinations.

		Accuracy	AUC	F1 score	Precision	Recall	MCC
Three models	Three slices	68.87	71.35	68.4	70.52	67.44	0.384
	2/4/5	69.1	71.2	69.14	69.83	69.49	0.388
	2/4/6	68.87	71.58	67.83	72.24	65.96	0.391
	2/5/6	67.74	71.95	67.04	70.02	65.19	0.361
	4/5/6	68.68	71.31	68.46	69.67	68.17	0.379
	50,100,150	69.43	70.31	66.22	**74.65**	60.72	0.403
Five models	5 slices	69.62	71.42	68.72	72.47	66.72	0.403
	3slices + 2/4	69.81	71.28	69.71	71.37	69.32	0.404
	3slices + 2/5	69.81	71.35	69.71	71.33	69.32	0.404
	3slices + 2/6	70	71.47	69.98	71.33	69.69	0.407
	3slices + 4/5	**70.19**	71.64	70.06	71.55	69.69	**0.411**
	3slices + 4/6	70	72.18	70.05	71.13	70.06	0.407
	**3slices + 5/6**	69.62	72.5	70.12	70.11	**70.81**	0.397
	3slices + 50,150	69.81	**72.83**	68.25	73.08	65.22	0.406
Six models	3slices + 2/4/5	70	72.04	**70.19**	71.56	70.45	0.41
	3slices + 2/4/6	69.81	72.36	69.98	71.45	70.08	0.406
	3slices + 2/5/6	68.87	72.75	69.23	69.75	70.07	0.386
	3slices + 4/5/6	68.87	72.62	68.6	70.35	68.57	0.387

**Notes.**

Values in bold indicate the best-performing model for each evaluation metric.

First, when three models were assembled, using predictions of three multi-scale networks achieved the best accuracy of 69.43% and exhibited a high increase from the individual models at 67.55%, 67.93%, and 66.04%. Next, the ensemble of five models was compared between predictions of five slices, three slices and two multi-kernel networks (*e.g.*, multi-kernel networks of kernel size 2 or 4), and predictions of three slices and two multi-scale networks. The results indicated that assembling five slices offered slightly higher performance than assembling three slices. The AUC and precision had the highest value of 72.83% and 73.08%, respectively, when the predictions of three slices and two multi-scale networks were assembled. Furthermore, the best combination was achieved by using predictions of three slices and two multi-kernel networks, 5 × 5 and 6 × 6, as this combination displayed noticeably higher performance on the F1 score and recall at 70.12% and 70.81%, respectively, which were essential evaluation metrics in clinical prediction tasks. Lastly, the results of six models, predictions of three slices, and three multi-kernel networks were concatenated. The results demonstrated that an ensemble of three slices and 2 × 2, 4 × 4, and 5 × 5 multi-kernel networks achieved the best performance but did not significantly improve over the previous ensemble of five models. Moreover, the ensemble of five models presented a better overall MCC than the ensemble of six models, indicating higher prediction quality.

### Activation mapping of deep-learning networks

**Figure 5:**
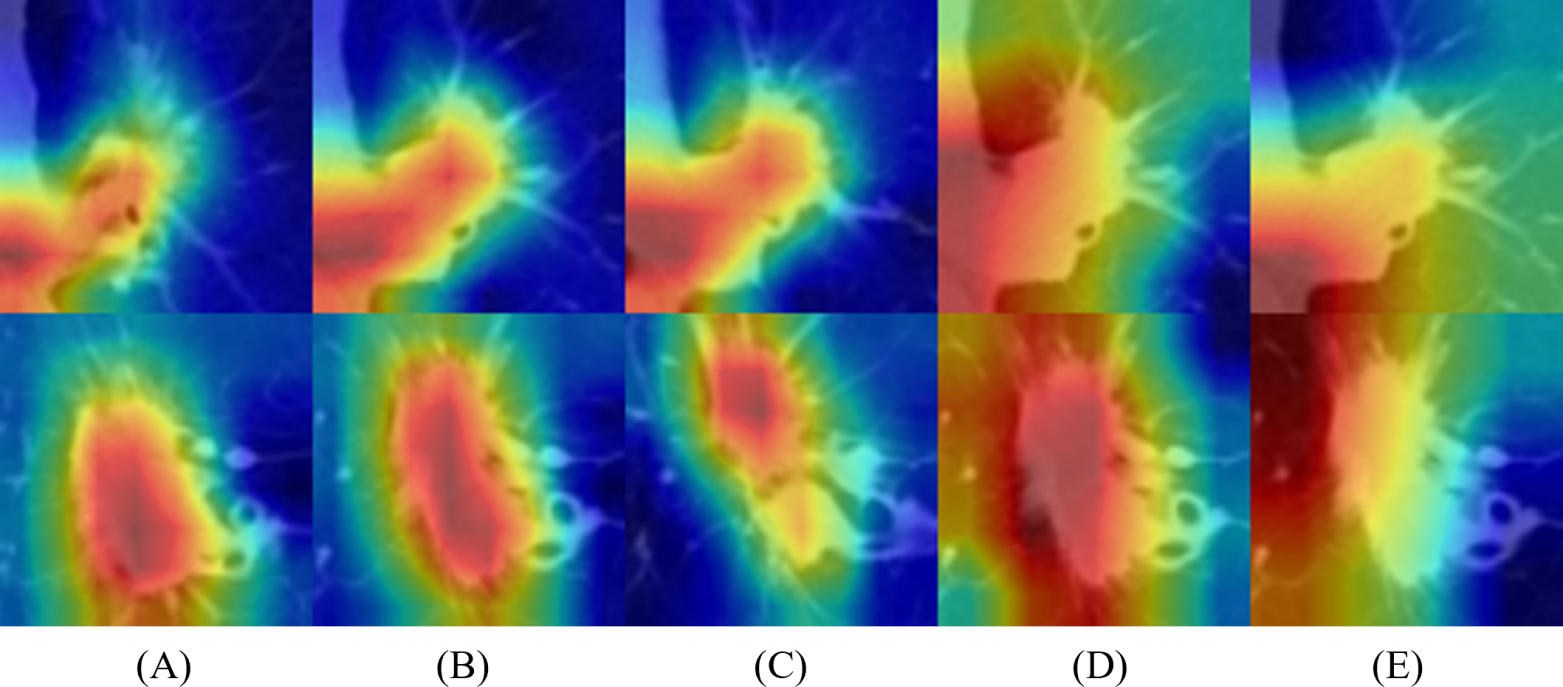
Merged images of tumor and gradient class activation maps (Grad-CAM) of two patients: results of 2D-CNN models based on (A–C) three slices and (D, E) two multi-kernel networks, 5 × 5, and 6 × 6.

To better understand the behavior of the CNN, we visualized the network activation maps over the final convolutional layer of the model, based on three slices and 5 × 5 and 6 × 6 multi-kernel networks (Fig. 5). The heatmap represents the contribution of each region on image classification using color from red to blue. Each row represents the merged images of input and the gradient class activation map of one patient in the order of three slices and a medium slice with two multi-kernel networks. We observed a significant difference in the regions that influenced the most predictions, but this depended on the model architecture. For instance, the multi-kernel network predictions were primarily relevant to the peritumoral regions; whereas the intra-tumoral region was crucial for other models. However, the patients could not be predicted correctly with only three slices, indicating that assembling multi-kernel networks improved performance.

**Figure 6:**
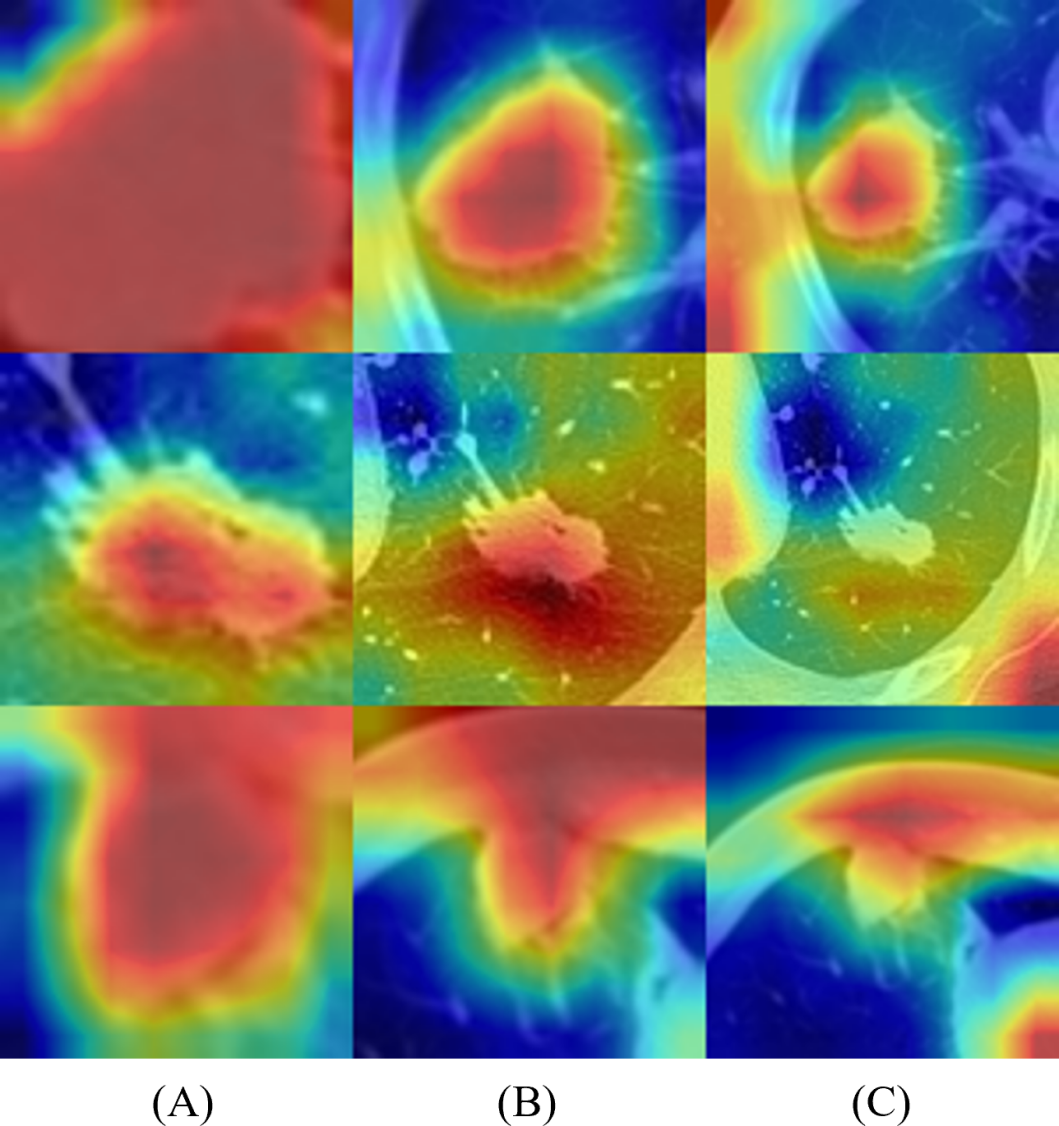
Merged images of tumor and Grad-CAM for three patients: three multi-scale network results: (A) 50 × 50, (B) 100 × 100, and (C) 150 × 150.

Figure 6 represents the results of Grad-CAM (Selvaraju et al., 2017) of multi-scale networks, which are in the order of 50 × 50, a medium slice of 100 × 100, and 150 × 150. The result reveals that the tumor is significant in the 50 × 50 input image and provides information on the texture and shape of the tumor. As for the larger scales, the surrounding tissues grew more significantly, enabling the network to capture local features and global information from the surrounding tissues.

### Benchmark study comparison

The prediction performance of the benchmark studies was obtained under the same cross-validation setting for proper comparison. Tables 4 and 5 present the results of the benchmark studies and the proposed models for the proposed 2D CNN and ensemble model, respectively. Table 4 presents the number of trainable parameters and experimental results of the benchmark studies on the same medium slice tumor dataset. This study was benchmarked against a previous study that predicted head and neck cancer outcomes according to CT images (Diamant et al., 2019). In the benchmark study, only the tumor region annotated by the mask was used from the central slice of the 3D CT volume. Although it leveraged the same slice, the proposed model used a cropped image as input, which helped the model to capture information from the tumor periphery. For the benchmark study, batch normalization layers were employed after every convolutional block, and random shifting was removed to reduce the risk of over-fitting the dataset. Table 4 reveals that the proposed 2D CNN models for several inputs significantly outperform the benchmark model. Furthermore, we experimented with VGG16, VGG19, and ResNet18 as the pre-trained CNN benchmark models. The pre-trained CNN requires an input size of 224 × 224, thus we used bicubic interpolation for resizing. The same grayscale images were used for three channels to match the input size of the ImageNet pre-trained CNN. The three channels help the fine-tuned network to use all weights and exploit all learned information when extracting features from the pre-trained network (Paul et al., 2018). For all pre-trained networks, the deep features after the feature learning layers were extracted and went through three fully connected layers of 1000, 200, 1 unit, and sigmoid activation for the final prediction. Moreover, two dropout layers with 0.5 probability were applied after the linear layers to prevent overfitting. The models were trained for 300 epochs, and the same learning rate approach was used as the proposed 2D-CNN. Although the pretrained CNN can be trained with little parameter and hyperparameter tuning, the proposed 2D-CNN model obtained significantly higher accuracy, AUC, and precision results. We surmise that the proposed model has a simple and effective CNN architecture, which is more suitable for relatively small medical datasets.

**Table 4:** Results of 2D-CNN models from the benchmark and proposed models on the same patient cohort.

Methods	Accuracy	AUC	F1 score	Precision	Recall	Trainable parameters
Diamant, André, et al. Diamant et al., (2019)	64.71	69.57	60.76	**69.3**	55.42	1M
Pretrained VGG16	63.02	66.35	66.75	60.12	75.69	40M
Pretrained VGG19	64.15	67.06	66.51	61.69	73.06	45M
Pretrained ResNet18	61.13	69.58	**69.69**	56.87	**90.1**	11M
**2D CNN (ours)**	**67.93**	**71.59**	68.13	68.97	68.95	94K

**Notes.**

Values in bold indicate the best-performing model for each evaluation metric.

**Table 5:** Results of ensemble models from the benchmark and proposed methods on the same patient cohort.

Methods		Accuracy	AUC	F1 score	Precision	Recall
Afshar et al. (2020a)	First scale	64.52	64.52	66.43	63.61	63.61
	Second scale	63.39	63.39	65.14	62.72	62.72
	Third scale	62.64	62.64	64.16	62.23	62.23
	3D-multiscale CapsNet	65.66	65.66	68.17	64.23	64.23
**Ours**		**69.62**	**72.5**	**70.12**	**70.11**	**70.81**

**Notes.**

Values in bold indicate the best-performing model for each evaluation metric.

A capsule network (CapsNet) (Sabour, Frosst & Hinton, 2017) is a deep learning architecture that can effectively model spatial relationships. The benchmark study in Table 5 proposed a 3D multi-scale CapsNet model, which took 3D patches of the nodules at three scales: 80 × 80, 100 × 100, and 120 × 120 to predict the nodule’s malignancy (Afshar et al., 2020a). The 3D input is composed of three slices: the central slice and two immediate neighbors. The output vectors of three CapsNets were concatenated and processed through a multi-scale model, consisting of a set of fully connected layers, similar to the proposed multi-model ensemble approach. The results demonstrated that the ensemble method through deep learning assisted in enhancing the benchmark model’s performance and improving the F1 score of each model from 66.43%, 65.14%, and 64.16% to 68.17%. Three 2D slices keep a smaller number of features for the network and reduce the graphics processing unit (GPU) memory usage compared with using the full 3D volume (Xu et al., 2019). The results imply that the proposed model performs better because it further reduces the training time and prevents the model from over-fitting. In addition, the training speed was significantly improved compared with this benchmark study, which used a 3D input. This outcome indicates that an ensemble of 2D CNN models is better than 3D CNN models when employed with a small dataset.

Majority voting and machine learning algorithms are commonly used to ensemble multiple features to make a final prediction in the medical field (Chennamsetty, Safwan & Alex, 2018; Cho & Won, 2003; Zhou et al., 2021). Regarding the best combination of models (ensemble of three slices and two multi-kernel networks of 5 × 5 and 6 × 6), majority voting and multiple machine learning algorithms were assessed to compare the evaluation metrics with the proposed deep learning-based multi-model ensemble method, listed in Table 6. Although the Gaussian Naive Bayes model exhibited the best performance, the proposed ensemble network performed better in all metrics. The results may be inferred from the fact that the neural network considers non-linear relationships of various models more effectively than other models (Xiao et al., 2018).

**Table 6:** Performance of various ensemble methods, machine learning algorithms, majority voting, and the proposed deep learning-based ensemble.

Ensemble Methods	Accuracy	AUC	F1 score	Precision	Recall
RF	67.36	69.95	67.47	68.4	67.43
LR	68.49	71.43	68.47	69.59	68.57
SVM	67.55	69.63	67.46	68.91	67.05
GB	66.23	70.05	66.18	67.46	65.93
**GNB**	**68.87**	**71.54**	**68.98**	**69.84**	**69.32**
LDA	68.11	71.18	68.04	69.37	67.82
QDA	67.92	67.67	67.96	69.37	67.44
Majority Voting	**68.87**	68.91	**68.98**	**69.84**	**69.32**
**Deep learning-based ensemble (Ours)**	**69.62**	**72.5**	**70.12**	**70.11**	**70.81**

**Notes.**

Values in bold indicate the best-performing model for each evaluation metric.

## Discussion

We proposed a novel deep learning-based ensemble model for NSCLC recurrence prediction. We trained multi-kernel and multi-scale networks on various input images and concatenated the prediction results. Input images include five slices at 5-mm intervals and two multi-scale images of 50 × 50 and 150 × 150. Multi-kernel networks are 2D-CNN models, which are applied on the central tumor slice, using convolutional kernel sizes of 2 to 6. Furthermore, multi-scale networks use various multi-scale inputs, which depend on the extent to which the tumor surroundings are included. Following the 2D CNN models, the deep learning-based ensemble approach was employed for the newly created dataset for final prediction. We compared the proposed ensemble model and individual 2D CNN models trained in various settings.

The 2D CNN models performed better on five slices and multi-kernel networks than on the two multi-scale networks, especially in accuracy, F1 score, precision and MCC. Multiple ensemble models were compared to determine the best combination of 2D CNN models. We observed that using predictions of three slices and two multi-kernel networks of 5 × 5 and 6 × 6 offered the highest performance with smaller model ensembles. Additionally, the custom 2D CNN model performed better than the pre-trained CNNs because of its simple and effective CNN architecture, more suitable for small medical datasets (Table 4). According to the Grad-CAM results, the multi-scale and multi-kernel networks make predictions based on different image regions, which vary depending on the scales and kernel sizes. This observation agrees with the rationale of multi-scale input discussed in (Afshar et al., 2020a; Afshar et al., 2020b; Dou et al., 2018), showing that the tumor region, surrounding tissue, location, and attachment to the vessels, may be an informative area for time-to-event outcome prediction. Furthermore, the results imply that the deep learning-based ensemble approach offers a better prediction than a single model and outperforms various representative machine learning models and the majority voting algorithm. This outcome might be attributed to the fact that deep learning allows the model to leverage various features by effectively considering non-linear relationships. Moreover, the multi-scale input and multi-kernel networks may enable the ensemble model to learn multi-level contextual features (Xu et al., 2020). Although it is essential to crop the CT images into 2D patches from various slices and scales, the proposed method can provide the network with various information to learn and keep the number of features lower than fully 3D approaches. In addition, the proposed method can prevent the model from overfitting and reduce GPU memory usage and training time.

This study has the following limitations: We combined the VHS Medical Center dataset with the publicly available dataset and applied five-fold cross-validation to compensate for the small data size. Therefore, there was a lack of external validation because the number of training and testing cases was limited. Various CT scanners were used first, and we found that this method lowered the overall performance due to CT scanner variability. This result led us to only use CT images from two vendors: GE and SIEMENS. Future studies could incorporate all vendor types to improve the prediction results and reduce inter-scanner variability. For instance, the style transfer techniques, such as multimodal unsupervised image-to-image translation and domain-specific mappings (Chang, Wang & Chuang, 2020; Huang et al., 2018), could be used to match the smoothness or sharpness of the CT images. The proposed ensemble framework could also be applied to various clinical prediction tasks to effectively integrate features from multiple models.

## Conclusions

This study presents a deep learning-based multi-model ensemble network for predicting NSCLC recurrence in lung CT scans. Most studies on deep learning-based cancer prediction using CT images have focused on the features extracted from a single 2D slice or 3D volume. However, studies have shown that an ensemble approach with multiple models improves prediction performance. We analyzed the performance of 2D-CNN models using multi-scale inputs and multi-kernel networks, and various combinations were evaluated to determine the best combination for the final ensemble model. The fusion of 2D-CNN models, using three slices and two multi-kernel networks, 5 × 5 and 6 × 6, provided the best performance with an accuracy of 69.62%, AUC of 72.5%, F1 score of 70.12%, and recall of 70.81%. Thus, the proposed network successfully captures information from various models. These promising results allow the proposed scheme to be used as a decision aid for patients with NSCLC by selecting patients who may benefit from adjuvant therapies.

## Additional Information and Declarations

### Data Availability

The following information was supplied regarding data availability: The clinical and CT image dataset is available at OSF: Choi, Jang-Hwan. 2022. “LDCT.” OSF. November 15. doi:10.17605/OSF.IO/TWK96. The source code is available at GitHub and Zenodo:
https://github.com/gyyeon/NSCLC_prediction/. gyyeon. (2023). gyyeon/NSCLC_prediction: v1.0 (v1.0). Zenodo. https://doi.org/10.5281/zenodo.7644809.
